# Gender stereotypes and their correlates: the moderating role of voluntary sports club membership

**DOI:** 10.3389/fpsyg.2023.1236439

**Published:** 2023-09-07

**Authors:** Pamela Wicker, George B. Cunningham

**Affiliations:** ^1^Department of Sports Science, Bielefeld University, Bielefeld, Germany; ^2^Laboratory for Diversity in Sport, Department of Sport Management, University of Florida, Gainesville, FL, United States

**Keywords:** gender, role attributes, Europe, voluntary sports club, women

## Abstract

**Introduction:**

This study examined the correlates of gender stereotypes and the moderating role of membership in a voluntary sports club. Drawing on the contact hypothesis, this study argues that gender stereotypes are lower when individuals regularly have the opportunity to meet and play sport with such people, for example in a voluntary sports club.

**Methods:**

Survey data from the European Values Study are used for the analysis (*n* = 36,185; 30 countries). Gender stereotypes are measured with statements on gender role attributes.

**Results:**

Regression results show that membership in a voluntary sports club, being a student, income, and living in a more gender equal country significantly reduce gender stereotypes. On the contrary, male gender, living in a partnership, having children, lower and medium education, part-time employment, self-employment, unemployment, being a home maker, and living in a small town are correlates of higher gender stereotypes. Interacting the latter correlates with sports club membership support its moderating effect in the sense that most correlates turn insignificant or have smaller coefficients. The only variables retaining their coefficient size are self-employment and living in a small town.

**Discussion:**

The findings support the contact hypothesis and suggest that sports clubs are places that lessen gender stereotypes.

## 1. Introduction

Stereotypes reflect the set of characteristics and behaviors people consider representative of a social group, or even people presumed to be members of that group (Stangor, 2016). Stereotypes are the pictures that come to mind when thinking about a group of people (Lippman, 1922). Though they can help when needing to make estimates quickly, in other cases, gender stereotypes have the potential to cause harm by reinforcing boundaries and limiting opportunities (Ellemers, 2018). Understanding the factors that shape the perception of gender stereotypes is important because gender stereotypes can be formed early. For example, children as young as 6 endorse gender stereotypes about intellect, as girls are less willing than boys to consider members of their gender as “really, really smart” (Bian et al., 2017, p. 389). These beliefs correspond to the activities in which girls and boys engage (Bian et al., 2017). In adolescence, girls and boys continue to endorse stereotypical gender attitudes, with their family, peers, and (to a lesser extent) media and schools all serving to shape these beliefs (Kågesten et al., 2016). Finally, gender stereotypes permeate virtually all aspects of society, including advertisements (Åkestam et al., 2021), educational training and professional development (Myers et al., 2020), customer service interactions (Otterbring et al., 2021), work opportunities (Chang and Milkman, 2020), and salary negotiations (Pardal et al., 2020). These examples show how manifest gender stereotypes are within society and how early they are developed.

Importantly, stereotypes are socially constructed and therefore malleable. A Spanish study offers an illustration (Garcia-Retamero et al., 2011): The authors examined perceptions of women and men in the past, present, and future, and found that study participants considered women and men more similar now than in the past, and they anticipated further similarities in the future. Other researchers have focused on factors that might change people’s stereotypes, such as interventions, with a meta-analysis of these studies showing that stereotypes can be changed (Lenton et al., 2009). Strategies focusing on examples of people who did not fit the stereotypical mold (i.e., a heterogeneity approach) were particularly effective. In other cases, researchers have examined societal factors and their role in shaping stereotypes. For example, in a study of 66 countries, a negative association between the share of women pursuing a degree in science and both implicit and explicit gender-science stereotypes was found (Miller et al., 2015). In another examining gender stereotypes among Germans and Nigerians, differences in how people thought of women as communal and men as agentic were evident (Obioma et al., 2022).

This research suggests that gender stereotypes are pervasive and can influence opportunities and experiences for women and men; yet, stereotypes can change, whether through intentional interventions or variations in societal factors. Therefore, it is important to understand the correlates that shape gender stereotypical perceptions in the population and get insights on factors that have the potential to alleviate such perceptions. Existing research has typically focused on a few select factors, such as individuals’ social status (e.g., Rowley et al., 2007), age (e.g., Barreto and Doyle, 2023), employment situation (e.g., Kucinskas and van der Does, 2017), marital status (Cunningham et al., 2005), or living area (e.g., Deole and Zeydanli, 2021). While existing research has provided valuable insights on select factors, the body of knowledge would benefit from including multiple factors within one study. Given that multiple factors are at work in shaping gender stereotypes, such research would enhance our understanding of the myriad of correlates.

Seeking to extend this understanding, the purpose of this study was to examine the correlates of gender stereotypes and the moderating role of voluntary sports club membership. Previous research examining gender stereotypes in sports contexts has focused on gender differences in the type of sports selected by women and men, the perceived gender appropriateness of sports, socialization and gender identities in sports (Chalabaev et al., 2013), expectations of sporting success and the value placed on success, and the role of parents shaping sporting ambitions of girls and boys (Boiché et al., 2013). The context where sports takes place and the role of different sports institutions in shaping gender stereotypes has not yet been examined.

The study advances the following two research questions: (1) what factors are correlated with the perception of gender stereotypes by European residents and (2) which of these relationships are moderated by membership in a voluntary sports club? In the theoretical part, ten hypotheses will be developed about individual and societal correlates of gender stereotypes. These factors speak to the first research question. Moreover, drawing on the contact hypothesis, we suggest that voluntary sports clubs serve as places where members can meet, play, and socialize with members of other sociodemographic groups, especially women, hence reducing gender stereotypes. This theoretical discussion results in the last hypothesis proposing a moderating role for sports club membership and speaking to the second research question. The research questions are answered using survey data from a multi-country European study.

The rationale of the study is to identify individual and societal antecedents of gender stereotypical perceptions of European residents and if sports club membership represents a moderating factor in the sense that it alleviates the perceptions of gender stereotypes. The results of regression analyzes revealed several correlates of gender stereotypes and a moderating effect of voluntary sports club membership in the sense that it reduces the level of stereotypes for several correlates. The findings contribute to the literature on gender stereotypes by explicitly measuring gender role attributes and identifying characteristics of individuals with higher and lower perceptions of gender stereotypes. The present approach is innovative because existing studies on gender stereotypes have only looked at selected correlates, neglecting the whole portfolio of individual and societal factors. Another contribution relates to the identification of a moderating factor which reduces the impact of several stereotype-raising factors. This contribution is innovative as existing studies have focused on gender stereotypes associated with specific sports (Chalabaev et al., 2013), neglecting the role of the institutional context where sports is offered and played. Examining the role of non-profit sports clubs which represent an important sports provider in Europe is critical for understanding for understanding their societal relevance.

## 2. Conceptual framework and hypotheses

### 2.1. Antecedents of gender stereotypes

We conceptualize multilevel factors as potential antecedents of people’s gender stereotypes. This position is consistent with the perspective that people’s personal characteristics and experiences, and societal factors all shape their attitudes and beliefs (Cunningham, 2019). Importantly, the review focuses on research examining gender stereotypes and the perceived roles of women in society. It excludes studies on sexuality in sport (Anderson et al., 2016) and sexual prejudices (Baiocco et al., 2020).

#### 2.1.1. Background and experiences

Previous researchers have shown that people’s background and experiences are related to the gender stereotypes they maintain. Social status helps explain some of these differences, as people will generally express bias in favor of their own group (Crocker and Major, 1989). Furthermore, high status people might be more likely to endorse traditional stereotypes about people not like them, relative to those beliefs that promote their own groups (Rowley et al., 2007). From this perspective, as men frequently have higher social status than do women, they might also be more likely to endorse gender stereotypes. For example, in a large-scale study out of New Zealand, men expressed more sexist attitudes than did women, and these differences at least partially manifested from men also endorsing more gender-specific systems justification than women (Sibley and Becker, 2012). Relatedly, in multiple studies out of the US, Grubbs et al. (2014) found that men’s expression of sexist beliefs were predicted by their psychological entitlement, or the ensuring attitudes of deservingness and demandingness. Given these patterns, we predicted:

*Hypothesis 1*: men will express stronger gender stereotypes than will women.

We also explored the influence of age. Across a host of social issues, younger generations adopt more progressive and welcoming perspectives than do their older counterparts. These patterns hold for gender stereotypes, too. Illustrative of such, Bhatia and Bhatia (2021) used machine learning to examine gender stereotypes over the course of the 20th century. They found that the strength of the biases had diminished. These changes were due to changes in how people thought about stereotypically feminine traits. These findings are consistent with Barreto and Doyle’s review of the literature, where they observed that older people generally express more biased attitudes toward women, relative to their younger counterparts (Barreto and Doyle, 2023). Thus, we predicted:

*Hypothesis 2*: age will be positively associated with gender stereotypes.

Marital status and the presence of children might link with individuals’ gender role attitudes. Relative to their peers, people who hold traditional gender attitudes might be more likely to enter into marriage or a registered partnership, and further, they are potentially more inclined to have children (Goldscheider and Goldscheider, 1992). Both activities align with traditional views of women, men, and their relationships with one another. Consistent with this view and drawing on a 31-year panel data study, people who expressed more egalitarian gender attitudes were more likely to live independently and less likely to have children in marriage. The patterns held for women and for men (Cunningham et al., 2005). Other researchers have found that entering into marriage or having children might shape people’s gender stereotypes: Endendijk and colleagues followed parents for 4 years after their youngest child turned age 1 and found that parents’ implicit gender stereotyping and behaviors became more conservative over time. The only exceptions were among men who already held such views and among older, highly educated women (Endendijk et al., 2018). Based on this collective research, we predicted:

*Hypothesis 3*: people who are married or in registered relationships will be more likely to express gender stereotypes than their peers.

*Hypothesis 4*: people with children will be more likely to express gender stereotypes than their peers.

Socioeconomic status, including people’s education, employment, and income, might also play a role. First, education commonly promotes ideas of equal opportunities and egalitarian norms. A study of attitudes toward foreigners among Europeans from 1988 to 2008 (Gang et al., 2013) showed that educational attainment was predictive of positive attitudes toward foreigners, leading the authors to describe education as a “strong antidote” (p. 177) for more exclusionary sentiment. Second, most educational institutions are co-educational, and as such, women and men have a chance to interact regularly with one another and observe how each other excel in the classroom and other educational endeavors. In line with these possibilities, a panel study from the UK and Switzerland examined gender attitudes in Europe following World War II, a timeframe that coincided with compulsory schooling implemented in many countries. Results showed that increases in educational attainment were related to more positive gender attitudes (Deole and Zeydanli, 2021). Based on this evidence, we predicted:

*Hypothesis 5*: educational attainment will be negatively associated with gender stereotypes.

The second component of socioeconomic status, employment, might also impact gender stereotypes. Researchers have shown that, particularly among women, homemakers have more traditional gender attitudes than do people who work outside the home (Leupp, 2019). In this case, people might endorse the societal expectations that women carry more of the childrearing duties than men and therefore, through their homemaking duties, further develop their traditional gender attitudes. On the other hand, people who work outside the home are likely to observe women working and excelling in the organization, dynamics that might prompt more egalitarian gender attitudes. Likewise, unemployment status might relate to more traditional gender attitudes. Unemployment can elicit feelings of uncertainty and stress, and as a way of compensating, some people turn to traditional perspectives and values. To illustrate, Shoss developed a conceptual model to demonstrate the impact of job insecurity (Shoss, 2017). She showed how insecurity represented a breach in the social contracts employees had with their employers, unions, and politicians. As a result, they were likely to cope by adopting more protective attitudes and approaches. These dynamics help explain the observed relationship between unemployment and traditional social and political attitudes (Diaz et al., 2011), including gender attitudes (Kucinskas and van der Does, 2017). Accordingly, we hypothesized:

*Hypothesis 6*: employment outside the home will be negatively associated with gender stereotypes.

We also predicted that the third element of socioeconomic status, income, would influence gender stereotypes. As education and the prestige of one’s job increase, so too does their income. Thus, given that we expected educational attainment (H5) and employment outside the home (H6) to relate negatively to gender stereotypes, income is likely to follow a similar pattern. Indeed, in a large-scale study of Australians, Perales and Bouma observed that high income earners were less likely than their peers to endorse patriarchal gender beliefs (Perales and Bouma, 2019). Thus, we predicted:

*Hypothesis 7*: income will have a negative association with gender stereotypes.

#### 2.1.2. Societal factors

Societal factors might also influence people’s gender stereotypes. This perspective is consistent with social ecological and multilevel views, whereby people’s beliefs are shaped by their personal factors (as previously outlined) as well as their social context. Several researchers have demonstrated these patterns. Rogers et al., for example, focused on boys’ gender beliefs during adolescence (Rogers et al., 2021). Drawing from developmental-context principles, they showed how boys’ relationships with peers, their behaviors, and the broader sociocultural context all served to shape the boys’ ideas about masculinity. Similarly, in her review, Ellemers noted that interactions among parents and children, the media, the language people use, body postures, and emotional expressions can all shape how people interpret gender stereotypes (Ellemers, 2018).

Community size is one factor that could relate to people’s gender stereotypes. From one perspective, in densely populated areas, people encounter a variety of people, observing them in different roles and engaging in different behaviors. This variability and the frequency with which it is observed might break down preconceived ideas about the acceptable roles for women and men. Relatedly, urban areas might be more likely than rural settings to preserve egalitarian values and norms. A study of gender stereotypes in Spain showed that people living in sparsely populated areas maintained gender stereotypes more strongly compared to their peers in more urban areas (Garcia-Retamero et al., 2011). Similarly, in an ethnographic study, Charles and Johns reflected on how growing up in rural Australia contributed to stereotypical ideas about gender and unintended sexist attitudes (Charles and Johns, 2022). Finally, a study of Europeans’ gender stereotypes indicated that highly educated women living in urban areas expressed more egalitarian attitudes than their peers living in more rural settings (Deole and Zeydanli, 2021). Based on this work, we predicted:

*Hypothesis 8*: community size will hold a negative association with gender stereotypes.

In the current study, we focused on a country’s record of gender equality. In more gender equal countries, it is expected that residents are more used to women being equally represented in all sectors of society and leadership positions. This representation leads to increased visibility of women and increased knowledge about women, ultimately reducing gender stereotypes. Though empirical examination of these possibilities is limited, there is some scholarship to support the notion that gender dynamics at the national level influence people’s beliefs and behaviors. For example, Nosek and colleagues analyzed data from 34 countries, assessing the implicit gender stereotypes at the country level. They found that stereotypes about women and men in science fields were predictive of gender differences in math and science achievement among adolescents (Nosek et al., 2009); thus, the national context influenced people’s individual outcomes. In research tangentially related to ours, Henry and Wetherell showed how countries with strong gender equality also had residents who expressed positive attitudes toward lesbian and gay people (Henry and Wetherell, 2017). Based on this collective evidence, we predicted:

*Hypothesis 9*: country-level gender equality will be negatively associated with gender stereotypes.

#### 2.1.3. The moderating role of voluntary sports clubs

Thus far, we have predicted that personal characteristics and societal-level factors might impact the gender stereotypes people endorse. We also suspect that membership in a voluntary sports club might influence these relationships. Sports clubs serve as spaces where community members can remain active and participate in sport throughout their lifetime, but they also serve broader societal functions, too. For example, they are considered schools of democracy given their non-profit status and the associated democratic processes how decisions are made (Breuer and Feiler, 2022). Many European countries are characterized by a dense network of voluntary sports clubs, meaning that residents across the country can be a member of such a club [for an overview see Breuer et al., 2015]. Sports clubs provide a variety of sport programs that cater for many different population groups, including older people, immigrants, people with health issues, and women (Lamprecht et al., 2017; Breuer and Feiler, 2022). Glancing at membership statistics indicates that across all sports, women represent about 30–40% of club members (Nichols and Taylor, 2015; Seippel and Skille, 2015; Breuer and Feiler, 2022). Collectively, voluntary sports clubs are places where members meet to play sport together, compete, gather at social events, engage in democratic processes, and contribute to club operations by engaging in voluntary work (Breuer and Feiler, 2022). Hence, members have many opportunities to regularly meet people from other social groups, including women.

From a contact hypothesis perspective, the positive interactions with people who are different can serve to reduce anxiety and improve intergroup relations (Allport et al., 1954; Pettigrew and Tropp, 2006). These benefits are likely to manifest when people cooperate with one another, have equal status, share common goals, and have institutional support for positive interactions (Allport et al., 1954). These conditions of contact are common in sport and among people on sport teams (Lee and Cunningham, 2014); thus, participation in voluntary sports clubs has the potential to offer those benefits. Although exceptions might occur, engagement in voluntary sports clubs can help people meet dissimilar others and develop unique forms of social capital (Auld, 2008). In further support of this position, Mousa examined the outcomes among Christians and Muslims who participated in soccer clubs in post-war Iraq (Mousa, 2020). The Christians had all been displaced by ISIS. One of the teams consisted of all Christians and the other had players from both religious traditions. Christians who played on religiously mixed teams expressed more inclusive and positive attitudes and behaviors than did their counterparts. They voted for Muslims for team awards, enlisted to play on mixed teams the following season, and continued in their athletic training with Muslims. These findings point to the potential for sport to help alleviate biases. Thus, we expected:

*Hypothesis 10*: participation in voluntary sports clubs will be negatively associated with gender stereotypes.

Beyond the direct effects, it is possible that participation in voluntary sports clubs might impact the effects of the personal and societal-level factors. In this case, membership in such entities would moderate the relationships between those factors and gender stereotypes (Cunningham and Ahn, 2019). For example, though we expect that people living in countries with high gender inequality to also express gender stereotypes, this relationship might be negated among people who are members of voluntary sports clubs. And though we present the possibility for the country-level factor, the same possibilities exist for the personal and background characteristics, too. Given this potential, we developed the following hypothesis:

*Hypothesis 11*: membership in a voluntary sports club will moderate the relationship between personal and background (H11a) and societal-level (H11b) factors and people’s gender stereotypes in the sense that it alleviates stereotype levels.

## 3. Methods

### 3.1. Sampling and data

The empirical analysis uses data from the 5th wave of the European Values Study (EVS). Data were collected between 2017 and 2020 in 34 European countries using face-to-face and computer-assisted web interviews. The questionnaire was provided in all languages that were spoken by at least 5% of the country’s population. Countries with more than 2 million inhabitants had a target sample of 1,200 respondents, while smaller countries aimed at 1,000 respondents. The target group were adult residents (i.e., 18 years and older) living in private households. The country-specific samples cover this target population. The combination of the above sampling methods (i.e., face-to-face and web interviews) was used to reach all population groups and ensure that the subsamples are representative of the adult resident population of the respective country. Previous research has provided evidence of the digital gap in the population: The extent of internet use and the types of internet activities differ depending on individuals’ socio-demographic characteristics, such as gender, age, education, and income (Drabowicz, 2014; van Deursen et al., 2015). Therefore, it is important to also employ non-web-based sampling methods like face-to-face interviews as these ensure that all population groups have the possibility to be included in the survey. The underlying questionnaire is the same in both sampling methods. Persons were randomly selected for the interviews and repeatedly contacted (EVS, 2023). Altogether, *n* = 56,491 individuals took part in the survey.

During the data cleaning, observations with missing values on relevant variables for this study were removed. Missing values were highest for the income question (~8,500), gender role attitudes (~6,300), voluntary sports club membership (~1,000), and number of children (~1,000). Overall, about 20,000 observations were removed because of non-response to some questions. Collectively, *n* = 36,185 respondents from 30 countries can be used for the empirical analysis. Table 1 provides an overview of the share and number of respondents by country and gender.

**Table 1:** Overview of respondents by country and gender in the sample (*n* = 36,185).

Country	Share of respondents (in %)	No. of respondents (*n*)	No. of women respondents (*n*)	No. of men respondents (*n*)
Albania	3.4	1,243	775	468
Azerbaijan	3.8	1,378	691	687
Austria	3.3	1,187	674	513
Armenia	3.7	1,341	730	611
Bosnia	3.9	1,415	797	618
Bulgaria	2.8	1,030	628	402
Belarus	3.2	1,160	682	478
Croatia	3.2	1,157	686	471
Czech Republic	2.7	992	591	401
Denmark	4.3	1,557	788	769
Estonia	2.5	921	617	304
Finland	2.2	808	420	388
France	4.2	1,536	843	693
Georgia	4.7	1,695	1,092	603
Germany	4.4	1,596	790	806
Hungary	2.9	1,067	621	446
Iceland	3.8	1,360	705	655
Italy	3.9	1,426	721	705
Lithuania	2.5	902	561	341
Montenegro	1.9	697	338	359
Norway	2.8	1,016	525	491
Poland	2.2	795	457	338
Romania	2.5	898	487	411
Russia	3.4	1,222	711	511
Serbia	2.6	940	528	412
Slovakia	2.4	853	517	336
Spain	2.3	825	462	363
Sweden	2.8	1,018	533	485
Switzerland	7.4	2,681	1,406	1,275
North Macedonia	1.8	666	334	332
Total	100.0	36,185	20,164	16,021

### 3.2. Questionnaire and variables

Table 2 shows the variables of this study. The outcome of interest is respondents’ gender role attitudes (Lomazzi, 2022) which are indicative of gender stereotypes and were assessed with seven questions. Respondents were asked to state their agreement on a four-point scale from 1 (disagree strongly) to 4 (agree strongly). Table 3 summarizes the items and their descriptive statistics. With a value of 0.861, Cronbach’s alpha is above the threshold of 0.7 (Hair et al., 2018), meaning the scale can be considered reliable. Therefore, a mean index was computed by adding up the items and dividing the score by 7.

**Table 2:** Overview of variables and descriptive statistics (*n* = 36,185).

Variable	Measurement	Mean	SD	Min	Max
Gender stereotypes index	Mean index of 7 items (Table 2; 1 = no stereotypes; 4 = very high stereotypes)	2.14	0.67	1	4
Sports club	Membership in a voluntary sports or recreation organization (1 = yes; 0 = no)	0.196	–	0	1
Man	Respondent’s gender (1 = man; 0 = woman)	0.443	–	0	1
Age	Age (in years)	49.18	17.30	18	82
Age2	Age squared (=Age*Age)	2717.83	1732.59	324	6,724
Partnership	Respondent is married or lives in a registered partnership (1 = yes; 0 = widowed, divorced, separated, never married)	0.563	–	0	1
Children_hh	Number of children in the household	0.75	1.00	0	4
Children_outside	Number of children outside the household	0.82	1.06	0	3
Edu_lower	Highest educational level is lower education (1 = yes; 0 = no)	0.181	–	0	1
Edu_medium	Highest educational level is medium education (1 = yes; 0 = no)	0.482	–	0	1
Edu_higher	Highest educational level is higher education (1 = yes; 0 = no)	0.338	–	0	1
Employed≥30 h	Employment 30 h/week or more (1 = yes; 0 = no)	0.422	–	0	1
Employed<30 h	Employment less than 30 h/week (1 = yes; 0 = no)	0.060	–	0	1
Self-employed	Self-employment (1 = yes; 0 = no)	0.061	–	0	1
Retired	Retired/pensioned (1 = yes; 0 = no)	0.249	–	0	1
Homemaker	Homemaker not otherwise employed (1 = yes; 0 = no)	0.053	–	0	1
Student	Student (1 = yes; 0 = no)	0.045	–	0	1
Unemployed	Unemployed (1 = yes; 0 = no)	0.089	–	0	1
Disabled	Not working because of disability (1 = yes; 0 = no)	0.015	–	0	1
Employed_other	Other employment (e.g., military service; 1 = yes; 0 = no)	0.007	–	0	1
Income	Household’s monthly net income (in TSD €; corrected for PPP)	2.319	2.029	0.094	12.507
Townsize<20 k	Living in town with less than 20,000 inhabitants (1 = yes; 0 = no)	0.712	–	0	1
Gender equality	Country’s gender equality index in 2016 (0 = perfect inequality; 1 = perfect equality)	0.734	0.053	0.669	0.874
Econ participation	Economic participation and opportunity of women (0 = perfect inequality; 1 = perfect equality)	0.703	0.063	0.574	0.823
Edu attainment	Educational attainment of women (0 = perfect inequality; 1 = perfect equality)	0.994	0.008	0.966	1.000
Health and survival	Health and survival of women (0 = perfect inequality; 1 = perfect equality)	0.972	0.010	0.939	0.980
Political empowerment	Political empowerment of women (0 = perfect inequality; 1 = perfect equality)	0.266	0.170	0.035	0.719
Volunteer	Volunteering in the last 6 months (1 = yes; 0 = no)	0.220	–	0	1
Not_nationality	Respondent does not have the country’s nationality (1 = yes; 0 = no)	0.033	–	0	1
Life satisfaction	All things considered, how satisfied are you with your life as a whole these days? (1 = dissatisfied; 10 = satisfied)	7.38	2.08	1	10
Health	All in all, how would you describe your state of health these days? (1 = very poor; 5 = very good)	3.74	0.94	1	5
Year	Year dummies for survey year (2017–2019)	–	–	–	–

**Table 3:** Items of the gender stereotypes scale reflecting gender role attributes (*n* = 36,185).

No.	Item (*1* = disagree strongly; *4* = agree strongly)	Mean	SD
1	When a mother works for pay, the children suffer	2.31	0.90
2	A job is alright but what most women really want is a home and children	2.47	0.92
3	All in all, family life suffers when the woman has a full-time job	2.42	0.93
4	A man’s job is to earn money; a woman’s job is to look after the home and family	2.15	0.98
5	On the whole, men make better political leaders than women do	2.04	0.94
6	A university education is more important for a boy than for a girl	1.64	0.76
7	On the whole, men make better business executives than women do	1.97	0.91
	Gender stereotypes index	2.14	0.67
	Cronbach’s alpha	0.861	

The main independent variable is membership in a voluntary sports club. Respondents were asked if they belong to a voluntary sports or recreation organization. Another predictor variable is respondents’ gender, which was assessed with the question ‘Are you a man or a woman?’. Possible answers were “A man,” “A woman,” “I do not know,” and “I prefer not to answer.” The resulting gender variable was binary coded with the categories man and woman. Age and its squared term were included to identify possible non-linear relationships between age and gender stereotypes. Another predictor is whether the respondent is married or lives in a registered partnership. Respondents were also asked to indicate the number of children that live in and outside their household. Highest educational level was assessed with different measures in each country and harmonized to ensure comparability, resulting in three dummy variables measuring lower, medium, and higher education. Respondents’ employment status was captured with a set of dummy variables, including employment of at least or less than 30 h per week; self-employment; retirement; being a home maker, a student, or being unemployed; not working because of a disability; and other employment. Monthly net household income was provided in purchasing power parities (*PPP*) to reflect differences in currencies and living conditions. Community size is measured with a dummy variable capturing town sizes of less than 20,000 inhabitants.

A country’s gender equality level was captured by a gender equality score, with a range from 0 (perfect inequality) to 1 (perfect equality). The value from 2016 is used to ensure adequate causality in terms of timing (World Economic Forum, 2016). The score consists of four dimensions, including economic participation and opportunity of women, educational attainment of women, health and survival of women, and political empowerment of women.

Finally, several control variables are included. First, we accounted for volunteerism, as previous studies have shown that gender can influence the types of volunteer activities in which people engage, as well as their experiences doing so (Helms and McKenzie, 2014; Gil-Lacruz et al., 2019). Respondents were asked if they volunteered in the last 6 months. Other researchers have suggested that immigrants might have different attitudes toward gender issues than do their peers (Pessin and Arpino, 2018). The respective dummy variable captures if the respondents do not have the nationality of the country where they live. Finally, previous studies have shown an association between gender beliefs and various dimensions of wellbeing. As such, we controlled for two aspects of wellbeing (Courtenay, 2000; Li et al., 2022): life satisfaction and health. The study also includes year dummies.

### 3.3. Data analysis

The empirical analysis strategy consists of three steps. First, descriptive statistics were obtained to give an overview of the sample. Second, linear regression analyzes were estimated with the gender stereotypes index as the dependent variable to answer the first research question. Model 1 includes the individual predictor variables from Tables 2 and a country’s overall gender equality score, while the four dimensions of this score were entered in Model 2. In preparation of the next step, the results were inspected for significant correlates that increase gender stereotypes. In a third step, another regression model with interaction terms was estimated to answer the second research question. Specifically, significant positive correlates from the first two models were interacted with sports club membership.

The independent variables were checked for multicollinearity using correlation analyzes and variance inflation factors (VIFs). All correlation coefficients were below the recommended threshold of 0.8 and the VIFs were clearly below the threshold of 10 (Hair et al., 2018). Therefore, the present regression models should not be distorted by multicollinearity. Typically, regression models of respondents from several countries would include country dummies. However, as these country dummies would be perfectly correlated with the five country-level gender equality variables, they cannot be entered into the regressions. An alpha-level of 0.05 was used for all statistical tests.

When examining topics like gender stereotypes which might have a subjective component, positionality and reflexivity of the researchers might be worth discussing. Positionality encompasses the relations with research participants, while reflexivity involves an understanding of how the process of doing research shapes the outcomes of the respective research. These aspects are more important to qualitative research than in in quantitative analysis (Corlett and Mavin, 2018). In the present study, the survey was not designed by the authors and the data were not collected by them; they were just analyzed as a secondary data source. Consequently, there was no contact between the researchers and the survey respondents. While the present empirical analysis and the underlying research questions were designed by the authors, the findings resulted from statistical analysis by employing established methods, leaving few room for being shaped by the authors. Given these points, positionality and reflexivity might be less of an issue in the present research.

## 4. Results

Table 2 displays the descriptive statistics. Respondents in the sample are on average 49.18 years old and 44.3% are male. Altogether, 3.3% do not have the nationality of the country they live in. More than half of respondents (56.3%) live in some form of registered partnership and have on average 0.75 children in the household and 0.82 outside of the household. Regarding the highest educational level, 18.1% have lower education, 48.2% medium education, and 33.8% higher education. Regarding employment, most respondents are employed at least 30 h per week (42.2%), followed by retirement (24.9%), unemployment (8.9%), self-employment (6.1%), and employment of less than 30 h per week (6.0%). Average monthly net household income is 2,319 (in PPP to account for country-specific differences in purchasing power). Regarding place of residence, 71.2% of respondents live in a town with less than 20,000 inhabitants. Average life satisfaction is 7.38 and subjective health is 3.75. Regarding leisure time engagement, 19.6% are a member of a sports club and 22.0% have volunteered in the last 6 months (Table 2).

The average perception of gender stereotypes is 2.14 (Table 2). Looking at the seven items (Table 3) suggests that the first three items capturing employment roles obtained the highest scores (2.31–2.47), while an educational item received the lowest score of 1.64. The countries where respondents live in have reached an average gender equality level of 0.734 (Table 2). Splitting this total score into four dimensions shows that countries score highest on women’s educational attainment (M = 0.994) as well as women’s health and survival (M = 0.972). Economic participation of women is ranked third with an average score of 0.703, while women’s political empowerment has a relatively low score with 0.266.

Table 4 summarizes the results of the regression analyzes. The models explain between 28.8% (Model 1) and 31.5% (Model 3) of the variation in gender stereotypes. In Models 1 and 2 excluding the interaction terms, membership in a voluntary sports club, being a student, income, volunteering, life satisfaction, and health are significantly and negatively associated with gender stereotypes, meaning that these characteristics lessen the perceived stereotypes level. Living in a more gender equal country in general and in a country scoring high on women’s educational attainment, health and survival, and political empowerment also reduces the perception of gender stereotypes significantly. Age has u-shaped relationship with gender stereotype perceptions, meaning that gender stereotypical perceptions decrease until a certain age and increase from this point onwards. The coefficients indicate that the turning point is 42 years.

**Table 4:** Linear regression analyzes for gender stereotypes index (*n* = 36,185).

Variable	Model 1	Model 2	Model 3
Constant	5.334***	7.332***	7.501***
Sports club	−0.063***	−0.051***	−0.310***
Man	0.183***	0.182***	0.174***
Age	−0.013***	−0.010***	−0.010***
Age2	0.000***	0.000***	0.000***
Partnership	0.079***	0.062***	0.055***
Children_hh	0.035***	0.023***	0.028***
Children_outside	0.019***	0.009*	0.012**
Edu_lower	0.152***	0.219***	0.207***
Edu_medium	0.094***	0.130***	0.121***
Edu_higher	Ref.	Ref.	Ref.
Employed≥30 h	Ref.	Ref.	Ref.
Employed<30 h	0.012	0.022	0.019
Self-employed	0.077***	0.055***	0.039**
Retired	0.007	0.008	0.007
Homemaker	0.127***	0.137***	0.132***
Student	−0.113***	−0.106***	−0.106***
Unemployed	0.048***	0.056***	0.051***
Disabled	−0.005	−0.004	−0.005
Employed_other	−0.028	−0.034	−0.036
Income	−0.052***	−0.042***	−0.043***
Townsize<20 k	0.049***	0.051***	0.037***
Gender equality	−3.837***	–	–
Econ participation	–	0.999***	0.951***
Edu attainment	–	−1.255**	−1.359***
Health and survival	–	−4.194***	−4.210***
Political empowerment	–	−1.456***	−1.453***
Volunteer	−0.037***	−0.042***	−0.043***
Not_nationality	0.067***	0.079***	0.080***
Life satisfaction	−0.022***	−0.019***	−0.019***
Health	−0.034***	−0.025***	−0.025***
Sports club × Male	–	–	0.042**
Sports club × Partnership	–	–	0.041*
Sports club × Children_hh	–	–	−0.025**
Sports club × Children_outside	–	–	−0.017*
Sports club × Edu_lower	–	–	0.061*
Sports club × Edu_medium	–	–	0.037*
Sports club × Employed<30 h	–	–	0.014
Sports club × Self-employed	–	–	0.092**
Sports club × Homemaker	–	–	0.033
Sports club × Unemployed	–	–	0.024
Sports club × Townsize<20 k	–	–	0.077***
Sports club × Econ participation	–	–	0.218
Year dummies (Ref. = 2017)	Yes	Yes	Yes
*R^2^*	0.288	0.314	0.315
*F*	563.238***	569.559***	404.983***

Displayed are the unstandardized coefficients; ***p < 0.001; **p < 0.01; *p < 0.05.

On the contrary, male gender, living in a partnership, having children in and outside of the household, having lower or medium education (as opposed to higher education), employment of less than 30 h per week, self-employment, being a home maker, and unemployment (as opposed to being employment at least 30 h per week), living in a small town with less than 20,000 inhabitants, and not having the country’s nationality are significantly and positively associated with gender stereotypes, indicating that these characteristics add to stereotypical perceptions about women in terms of gender role attitudes. Moreover, respondents living in a country with a higher economic participation and opportunities for women also have significantly higher stereotypical perceptions.

Model 3 includes the interaction terms for the examination of a moderating role of voluntary sports clubs. After accounting for the first order effects, membership in a sports club served as a moderator for several variables. Specifically, the effects of having children in and outside of the household changes signs and turns significantly negative. The previously positive associations of employment of less than 30 h per week, being a home maker, being unemployed, and living in a country with higher economic participation of women turn insignificant. The coefficients on male gender, living in a partnership, as well as lower and medium education become smaller, though remaining positive and significant. The only variables retaining their coefficient size and their significant positive effect are self-employment and living in a small town with less than 20,000 inhabitants. We summarize the findings and the support for our hypotheses in Table 5.

**Table 5:** Summary of findings and hypothesis testing.

Hypothesis – expected relationship with gender stereotypes	Result
1: Men – positive	Supported
2: Age – positive	Not supported
3: Marriage/registered partnership – positive	Supported
4: People with children – positive	Supported
5: Educational attainment – negative	Supported
6: Income – negative	Supported
7: Employment outside the home – negative	Supported
8: Community size – negative	Supported
9: Country-level gender equality – negative	Supported
10: Participation in voluntary sports club – negative	Supported
11a: Membership in a voluntary sports club – moderating effect for personal characteristics and alleviation of stereotype levels	Partially supported
11b: Membership in a voluntary sports club – moderating effect for societal-level variables and alleviation of stereotype levels	Partially supported

The empirical models also contain a set of control variables. The results for these variables show that respondents who have volunteered in the last 6 months have significantly lower levels of gender stereotypes. Moreover, the better the respondents’ health status and the higher their self-reported life satisfaction, the lower their perceived level of gender stereotypes. On the contrary, respondents who do not have the nationality of the country they live in scored significantly higher on gender stereotypes than individuals having the nationality of the country they live in.

## 5. Discussion

Gender stereotypes are pervasive and negatively impact women, their aspirations, and their opportunities. Importantly, however, stereotypes can also change, whether based on intentional interventions, contextual factors, or people’s life experiences (Lenton et al., 2009; Miller et al., 2015; Obioma et al., 2022). Drawing from this understanding, the purpose of the current study was to examine correlates of gender stereotypes and whether participation in voluntary sports clubs moderated these effects. Results show that membership in a voluntary sports club, being a student, income, living in a more gender equal country, volunteering, life satisfaction, and health significantly reduce gender stereotypes. On the contrary, male gender, living in a partnership, having children in and outside the household, lower and medium education, part-time employment, self-employment, unemployment, being a home maker, living in a small town, and not having the country’s nationality are correlates of higher gender stereotypes. Importantly, participation in a voluntary sports club is negatively associated with stereotypical gender beliefs and serves to offset otherwise stereotype-increasing effects of other factors. In the following sections, we discuss the findings, highlight the contributions, identify implications, note limitations, and suggest opportunities for future research.

### 5.1. Findings, contributions, and implications

Most of the hypotheses focusing on personal and background factors were supported (Table 5). Men, people who were married or in registered relationships, and people with children expressed more stereotypes than their peers, whereas people in higher social classes (i.e., high education, occupation, and income) expressed fewer stereotypes. These findings are consistent with our theorizing and previous scholarship in the area. The one exception was with respect to age, as we observed a u-shaped pattern. Thus, contrary to previous research on the topic (Bhatia and Bhatia, 2021; Barreto and Doyle, 2023), people in middle age are least likely to express gender stereotypes.

We also found that societal-level factors were related to people’s gender stereotypes – an area that has received comparatively less attention among scholars (Garcia-Retamero et al., 2011). The size of the community was one such element, as people living in communities with more than 20,000 people had more egalitarian gender beliefs. These findings align with the limited research in this area (Garcia-Retamero et al., 2011; Deole and Zeydanli, 2021) and suggest that urban communities might afford people more opportunities to interact with women and men in a variety of roles and contexts. As a result, stereotypes about typical women and men are likely curtailed. Similarly, country-level gender equality was related to more progressive gender beliefs. These findings align with previous research showing that as women and men within a particular country have equitable participation in society and life outcomes, that country’s residents adopt more egalitarian beliefs (Henry and Wetherell, 2017).

Finally, we found broad support for our contention that participation in voluntary sports clubs is linked with more progressive gender beliefs and can offset the impact of other factors that might spur gender stereotypes. These organizations play an important role in European countries (Breuer et al., 2015; Breuer and Feiler, 2022), cater to varied population groups, including older people, immigrants, people with health issues, and also women (e.g., Lamprecht et al., 2017; Breuer and Feiler, 2022), and are social spaces where women and men interact with one another (e.g., Breuer et al., 2015; Nichols and Taylor, 2015; Seippel and Skille, 2015). Our findings align with those in other contexts, where researchers have found that participation in sports clubs or on sports teams can influence people’s biases (Auld, 2008; Lee and Cunningham, 2014; Mousa, 2020).

Our study makes several contributions. First, we add to the growing body of research pointing to the efficacy of considering how factors at multiple levels of analysis relate to people’s diversity-related beliefs (Bond and Haynes, 2014; Ellemers, 2018; Cunningham, 2019). Indeed, focusing only on demographics, background variables, community factors, or societal factors would have only told part of the story. Instead, by incorporating a multilevel view, we identified a fuller picture of the factors shaping people’s gender stereotypes. Second, we identified a key moderator, participation in voluntary sports clubs. Moderators provide cues about when and under what conditions changes in correlating factors might occur, and in doing so, they help to expand theory (Colquitt and Zapata-Phelan, 2007). Thus, our study contributes to the theoretical understanding of gender stereotypes, when they might manifest, and their potential malleability.

Findings from the study also point to practical implications. First, the identification of correlates of gender stereotypes among residents in European countries is important to politics, gender policy, and the wider economy. The findings show which individuals are more likely to report stereotypes against women. Since the gender stereotypes items relate to gender roles at home as well as in politics, education, and the economy, they are relevant to understand the perceived role of women in several important domains of society. Therefore, policy makers can use these findings to develop gender policies and measures that raise awareness about the presence of stereotypes in the first place and reduce such stereotypical perceptions afterwards. Likewise, leaders in business enterprises and in education institutions can learn from this work by being sensitized for individual characteristics and conditions that nurture or prevent gender stereotypical perceptions among European residents.

Second, sport and exercise participation have many benefits, including those focusing on physical health, psychological wellbeing, and social health (Ruby et al., 2011; Eime et al., 2013; Nystoriak and Bhatnagar, 2018). There is also some evidence that sport participation can improve social relations among groups (Welty Peachey et al., 2020). Thus, in addition to the interventions Lenton and colleagues identified (Lenton et al., 2009), managers and policy makers seeking to reduce the prevalence of gender stereotypes can use sport. Again, drawing from the sport literature and contact hypothesis (Allport et al., 1954; Schulenkorf and Sherry, 2021), there is some evidence that sport participation is most likely to yield benefits when people cooperate, work toward a common goal, engage interdependently, and when institutional support for positive interactions is present. Schulenkorf and Sherry also noted the importance of sports being culturally relevant and exciting.

### 5.2. Limitations and future directions

This study has some limitations that can guide future research. It is limited to the available data and variables. Gender role attitudes were only assessed in the fifth EVS wave, meaning that the analysis could only be based on cross-sectional data. Therefore, the present analysis can only identify associations and not causal effects. In future research, it would be interesting to study with longitudinal data how becoming a sports club member might change gender stereotypes. While the present study included many correlates, gender stereotypes might be shaped by further factors, which were not assessed in the EVS and could therefore not be included in the present models. Future studies should also explore the effect of the frequency and intensity of club-based sport participation and how different sports and competitive settings shape gender stereotypes. Similarly, examining the role of club characteristics like club philosophy, size etc. might provide interesting insights that help to deepen our understanding of the moderating role of voluntary sports clubs as places where gender stereotypes can be alleviated. Combining the present study with another empirical study including more detailed club measures and also different indicators for gender stereotypes represents another avenue for future research. These indicators could include sexual orientation and sexual prejudices as these have been shown to be relevant in sports contexts, but are not included in the present study. Exploring the correlates of sexual orientation-related gender stereotypes would enhance our understanding of prevalent gender stereotypes in society and how they are shaped by sports participation and sports institutions like non-profit sports clubs.

## Data availability statement

Publicly available datasets were analyzed in this study. This data can be found at: https://europeanvaluesstudy.eu/methodology-data-documentation/evs-methodology/.

## Ethics statement

Ethical review and approval was not required for the study on human participants in accordance with the local legislation and institutional requirements. Written informed consent from the patients/participants or patients/participants’ legal guardian/next of kin was not required to participate in this study in accordance with the national legislation and the institutional requirements.

## Author contributions

PW and GC conceptualized and designed the study, wrote sections of the manuscript, and read and approved the submitted version. PW organized the data, conducted the data cleaning, performed the statistical analysis, and organized the submission and revision/resubmission process. All authors contributed to the article and approved the submitted version.

## Funding

We acknowledge the financial support of the German Research Foundation (DFG) and the Open Access Publication Fund of Bielefeld University for the article processing charge.

## Conflict of interest

The authors declare that the research was conducted in the absence of any commercial or financial relationships that could be construed as a potential conflict of interest.

## Publisher’s note

All claims expressed in this article are solely those of the authors and do not necessarily represent those of their affiliated organizations, or those of the publisher, the editors and the reviewers. Any product that may be evaluated in this article, or claim that may be made by its manufacturer, is not guaranteed or endorsed by the publisher.
